# Current and Future Therapeutic Approaches for Thymic Stromal Cell Defects

**DOI:** 10.3389/fimmu.2021.655354

**Published:** 2021-03-18

**Authors:** Alexandra Y. Kreins, Paola Bonfanti, E. Graham Davies

**Affiliations:** ^1^ Great Ormond Street Institute of Child Health, University College London, London, United Kingdom; ^2^ Department of Immunology, Great Ormond Street Hospital for Children NHS Foundation Trust, London, United Kingdom; ^3^ Epithelial Stem Cell Biology & Regenerative Medicine Laboratory, The Francis Crick Institute, London, United Kingdom; ^4^ Institute of Immunity & Transplantation, University College London, London, United Kingdom

**Keywords:** thymus transplantation, primary immunodeficiency, DiGeorge syndrome, severe combined immunodeficiency (SCID), regenerative medicine, PAX1, FOXN1

## Abstract

Inborn errors of thymic stromal cell development and function lead to impaired T-cell development resulting in a susceptibility to opportunistic infections and autoimmunity. In their most severe form, congenital athymia, these disorders are life-threatening if left untreated. Athymia is rare and is typically associated with complete DiGeorge syndrome, which has multiple genetic and environmental etiologies. It is also found in rare cases of T-cell lymphopenia due to Nude SCID and Otofaciocervical Syndrome type 2, or in the context of genetically undefined defects. This group of disorders cannot be corrected by hematopoietic stem cell transplantation, but upon timely recognition as thymic defects, can successfully be treated by thymus transplantation using cultured postnatal thymic tissue with the generation of naïve T-cells showing a diverse repertoire. Mortality after this treatment usually occurs before immune reconstitution and is mainly associated with infections most often acquired pre-transplantation. In this review, we will discuss the current approaches to the diagnosis and management of thymic stromal cell defects, in particular those resulting in athymia. We will discuss the impact of the expanding implementation of newborn screening for T-cell lymphopenia, in combination with next generation sequencing, as well as the role of novel diagnostic tools distinguishing between hematopoietic and thymic stromal cell defects in facilitating the early consideration for thymus transplantation of an increasing number of patients and disorders. Immune reconstitution after the current treatment is usually incomplete with relatively common inflammatory and autoimmune complications, emphasizing the importance for improving strategies for thymus replacement therapy by optimizing the current use of postnatal thymus tissue and developing new approaches using engineered thymus tissue.

## Introduction

Uniquely among hematopoietic stem cell derived lineages, T-cells require a second site for their development, namely the thymus. Thus, primary immunodeficiency disorders (PIDs) leading to T-cell deficiency may be a result of either hematopoietic lineage defects or defects of thymus stromal development or function. The latter includes athymia associated with DiGeorge syndrome (DGS), known as complete DGS (cDGS), and other monogenic defects resulting in failure of thymic stromal development. While hematopoietic stem cell transplantation (HSCT) from a matched family donor has been shown to promote survival in around two-thirds of cases of cDGS, an otherwise lethal condition, the quality of immune reconstitution was poor with low numbers of naïve T-cells and a restricted T-cell repertoire (1, 2). The survival after HSCT using alternative donors was very poor (1). It is logical that corrective therapies for these children should instead involve providing them with thymic tissue to facilitate the development of their normal hematopoietic precursor cells into mature T-cells. HSCT and thymus cell replacement treatments entered the clinic at very different times for both biological and historical reasons. The immunological function of the thymus was only understood four years after the first HSCT had already been reported in patients (3–5). HSCT research started as early as 1949 as a result of the recognition of the effect of radiations on the bone marrow. In addition, bone marrow is a liquid tissue and therefore relatively easy to harvest and to re-infuse with or without *ex vivo* treatment. Instead, the thymus is a primary lympho-epithelial organ, located in the thorax where access is difficult. The relatively late discovery in 1963 of the thymus as the site of T-cell development (5) resulted in a significant delay in our understanding of the thymic stroma. Furthermore, the complexity of its structure requires an interdisciplinary approach for consideration as to how to reconstitute its function. Transplantation of fetal thymus tissue to correct cDGS was first attempted more than fifty years ago but met with limited success (6–8). More recently the use of postnatal thymus tissue obtained from infant donors undergoing cardiac surgery for congenital heart disease was pioneered by Louise Markert at Duke University (9). Since that time, in more than one hundred cases treated at two centers worldwide, Duke University Hospital in the United States and Great Ormond Street Hospital in the United Kingdom, this has been shown to offer a better rate of survival and quality of immune reconstitution (10–12). This article will review the defects of thymus development focusing on those leading to athymia, the current approach to replacing thymus function through allografting with postnatal thymic tissue, and the future prospects for engineered thymus preparations with the ultimate aim of using a product of autologous origin.

## Thymus Development and Function

Thymus organogenesis has extensively been investigated in vertebrates, in particular in the mouse and chick. Early in embryogenesis a series of pharyngeal arches and pouches, known collectively as the pharyngeal apparatus, can be recognized. This includes inner endodermal and outer ectodermal regions associated with mesodermal tissue and neural crest cells (13, 14). From this structure, the thymus and the, developmentally closely allied, parathyroid glands develop, specifically in the region of the ventral part of the 3^rd^ pharyngeal pouch (15–17). This early development is under the control of multiple transcription factors including TBX1, HOXA3, PAX1, PAX9, EYA1, SIX1 and SIX4 (18, 19). Following this, FOXN1 becomes the predominant and enduring regulatory factor promoting thymus development, growth and maintenance of the thymus throughout life (20–22). These mechanisms seem largely conserved between mouse and human (23). From an early embryonic stage (7-8 weeks in humans) hematopoietic cells start to arrive in the thymus (23). The early thymus-seeding precursors originate initially from the aorta-gonad-mesonephros region (24–26) and later from the fetal liver (27) and the bone marrow. These thymus-seeding cells are necessary for the ongoing development of a functional thymus through a process of lymphostromal crosstalk (28, 29). The cellular interactions involved in embryonic thymic development have recently been studied in further detail using single-cell RNA sequencing in both mice (30) and humans (26, 31). The early thymic precursors maintain multipotency before differentiating down a T-cell committed pathway following interaction with NOTCH ligands (32) and other environmental signals.

The cellular composition of the thymus comprises hematopoietic cells and thymus stromal cells (31). The former are predominantly T-cells at various stages of development (thymocytes) but importantly, in addition to B-cells and macrophages, also include different types of dendritic cells (DC), some arising *de novo* in the thymus and others having migrated in (33). The antigen presenting capability of these DCs is very important in the process of negative selection as part of thymopoiesis (see below). Furthermore self-renewing lymphoid precursor cells have been identified in immunodeficient mice after transplantation of human thymus (34), suggesting the presence of lymphoid progenitors capable of long-term survival and T-cell development in postnatal human thymus. The thymus stroma comprises thymic epithelial cells (TEC), mesenchymal cells, fibroblastic cells and vascular endothelial cells. TECs are the hallmark cells of the thymus being classed broadly into two groups with distinct functions, cortical (cTEC) and medullary (mTEC). The cellular complexity of the thymic stroma with higher TEC heterogeneity is becoming increasingly evident (31, 35, 36). In the mouse, a common bipotent precursor for cTEC and mTEC compartments has long been described (37–39). Recent characterization of expanding human mTECs and cTECs, which lose their compartmental signature, is in keeping with this (35). Finally, the whole structure is supported by a scaffold of extracellular matrix (ECM), comprising, among other things, collagen and fibronectin, which facilitates thymocyte migration and cellular interaction (40–42). Microscopically, the thymus comprises three zones with distinct functions: subcapsular, cortical and medullary.

The process of thymopoiesis, the development of mature T-cells in the thymus, depends on serial steps taking place in distinct thymic microenvironments. This is well understood in the mouse and has been described elsewhere (29). Briefly, hematopoietic precursor cells leave the bone marrow and enter the thymus through the cortico-medullary junction and then pass into the cortex where they become committed to the T-cell lineage. The different stages of their development are characterized by the expression of cell surface molecules, including CD4 and CD8. The first thymic stage is the immature CD4^-^CD8^-^ double negative (DN) stage. Thymocytes then start to rearrange their T-cell receptors (TCRs), generating double positive (DP) thymocytes, expressing both CD4 and CD8. As they migrate from the cortex to the medulla, these DP thymocytes undergo positive and negative selection, respectively resulting in the elimination of non-reactive and self-reactive cells. Subsequently, the cells will egress from the thymus as functional single positive (SP) CD4^+^ or CD8^+^ recent thymic emigrants (RTEs) migrating to the periphery. While there are differences in T-cell development between humans and mice (43–46), the murine model continues to offer fundamental insight into this process and some of these developmental stages have been reproduced during the *in vitro* differentiation of human hematopoietic stem cells (HSCs) in co-culture systems with Notch ligand-expressing murine stromal cells (47–49). Additionally, novel molecular tools, such as high-throughput RNA sequencing, efficient high-precision genome editing and the possibility to use HSCs and induced pluripotent stem cells (iPSCs) from certain PID patients, are further facilitating the study of human T-cell development in new *ex vivo* (50–52) and *in vivo* models (53, 54).

**Figure 1:**
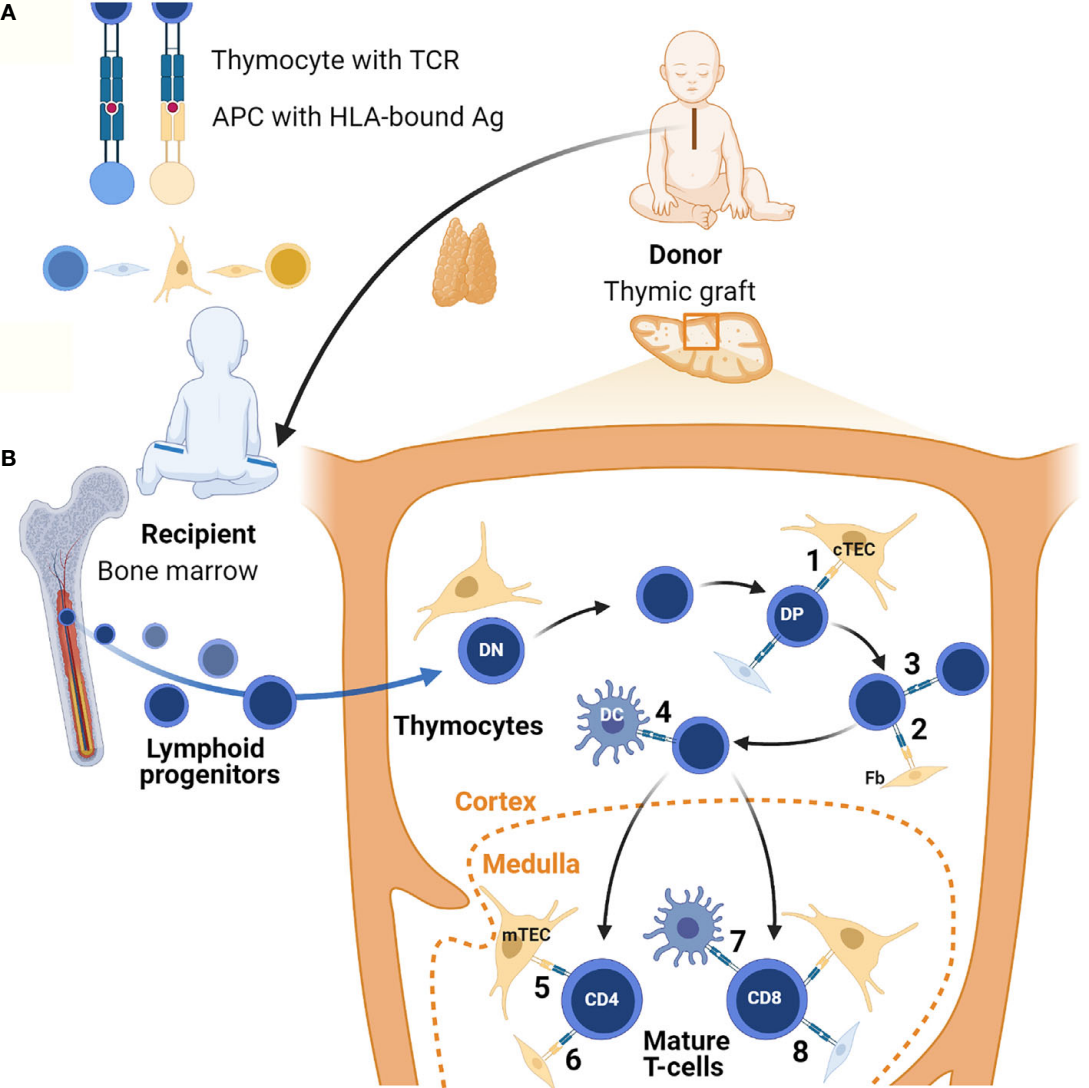
Lymphostromal crosstalk in thymic grafts. **(A)** Developing thymocytes communicate with antigen (Ag)-presenting cells (APC) through HLA-TCR interactions. APCs include both thymic stromal cells and hematopoietic cells. In thymic grafts these APCs can be host-derived (in blue) or donor-derived (in orange). The thymus tissue donor and the athymic patient are not HLA-matched. **(B)** After thymus transplantation, lymphoid progenitors migrate from the recipient’s bone marrow into the HLA-mismatched thymic graft. In the thymic cortex developing thymocytes undergo positive selection of competent T-cells through low-affinity engagement of their TCR with HLA-bound antigens. In the case of serendipitous, partial HLA-matching between donor and recipient, traditional antigen-presentation by cTECs may occur (1). Donor-derived fibroblasts (Fb) possibly also contribute to this (2). In theory, host-derived APCs of hematopoietic origin in the graft may also contribute to positive selection, for example through direct thymocyte-thymocyte interactions (3). Positively selected DP thymocytes then undergo negative selection to eliminate autoreactive T-cells. In the thymic graft this may also start in the cortex thanks to direct HLA-TCR mediated interactions with DCs of host origin (4). In normal thymus tissue generation of self-tolerance predominantly takes place in the medulla. If there is partial HLA-matching between the donor and the recipient, donor-derived mTECs and possibly fibroblasts can contribute to this in thymic grafts (5 and 6). Host-derived hematopoietic cells, in particular DCs, may also play a role (7). Additionally, it is possible that upon transplantation chimeric thymic stroma develops with stromal cells of host origin, both in the cortex and the medulla (8). Figure created with BioRender.com.

It is lymphostromal crosstalk consisting of MHC-TCR interactions between antigen-presenting cells (APCs) and developing thymocytes (**Figure 1A**), that supports thymopoiesis with positive and negative selection resulting in the generation of self-tolerant T-cells with a diverse TCR repertoire. During positive selection thymocytes undergo proliferation driven by their interaction with self-peptides expressed in association with major histocompatibility complex (MHC) molecules on cTECs and possibly other cells including thymocytes (55, 56) and fibroblasts (57, 58), though the physiological role of fibroblasts in this process has been disputed by others (59). Evidence suggests that the specificity of the MHC driving this is not critical to this process (60). The positively selected and expanded DP T-cells then undergo negative selection. During a first wave of negative selection, autoreactive DP thymocytes are eliminated in the cortex upon association with MHC-bound self-antigens presented by DCs (61, 62). Remaining thymocytes then mature into SP cells and migrate into the medullary zone where T-cell receptor rearrangement is completed. In the medulla, SP T-cells undergo a second, predominant wave of negative selection. Tissue restricted (self) antigens (TRAs) promiscuously generated in mTECs under the influence of the autoimmune regulator (AIRE) are presented to these T-cells in the context of self MHC expressed on the mTECs and DCs or on other cell types. T-cells with receptors recognizing self TRAs will either be deleted or develop down the pathway of regulatory T-cells (Tregs). Recently, a role for medullary fibroblasts has also been reported in central immune tolerance, specifically through lymphotoxin β-mediated signaling (63). Altogether, these processes lead to T-cell central tolerance to self in order to avoid autoimmunity.

## Abnormalities of Thymic Stromal Development and Function

Failure of development of the thymus can result from defects in the above described processes. The resulting immunodeficiency may vary from none through a mild infection susceptibility and predisposition to autoimmune disease to a severe combined immunodeficiency (SCID) picture with complete athymia and a susceptibility to life threatening opportunistic infections (64). There are a number of different causes of thymic hypoplasia and aplasia, including DGS and other monogenic defects, which are described in the section below and summarized in **Table 1**. Athymic patients have a profound T-cell lymphopenia and require life-saving corrective therapy.

**Table 1:** Disorders associated with congenital athymia.

Disorder	Etiology	N (%) TT	N Survival	N Autoimmunity
**cDGS**	**Chromosome 22q11.2 Deletion Syndrome ** **TBX1 deficiency** **TBX2 deficiency** **CHD7 mutations (CHARGE)** **Maternal diabetes** **Undefined**	30 (38%) 1 (0.01%) 1 (0.01%) 19 (24%) 12 (15%) 11 (14%)	53	AI thyroiditis: 17 AI cytopenias: 15
	**Total**	74 (94%)		
**T^-^B^+^NK^+^ SCID**	**FOXN1 deficiency** PAX1 deficiency (OTFCS2) Undefined	5 (6%) NA NA	4	0

Highlighted in bold are the defects for which the use of thymus transplantation (TT) has already been reported in a total of 79 patients (10–12, 65–71). Overall survival is of 72%. Outcomes regarding survival and autoimmune complications after transplantation are indicated in absolute numbers (N) of transplanted patients. The incidence of autoimmune (AI) complications is an underestimation given detailed information regarding long term clinical outcome is not available for all patients.

To date few genetic defects resulting in malfunction rather than failure of development of the thymus stroma have been identified. Deficiency of AIRE predisposes to multiple manifestations. Biallellic deleterious mutations in *AIRE* result in autoimmune polyglandular syndrome type 1 (APS1) also known by the acronym APECED (autoimmune polyendocrinopathy, candidiasis, ectodermal dysplasia) (72, 73). Hypoparathyroidism and adrenal insufficiency are the most common endocrinopathies, but APECED’s clinical picture is broader than previously recognized, including significant non-endocrine manifestations (74, 75). It has also been found that single allele *AIRE* mutations with a dominant-negative effect can lead to a milder predisposition to autoimmune disease (76, 77). Secondary AIRE dysfunction resulting in impaired central tolerance also plays a role in other PIDs, such as in patients with hypomorphic *RAG* mutations and *PRKDC* mutations (78–81). Similarly, impaired AIRE signaling has been proposed in early onset common variable immunodeficiency due to heterozygous *NFKB2* mutations, for which autoimmune features, including alopecia, hypopituitarism and serum autoantibodies, have been reported alongside a susceptibility to infections and hypogammaglobulinemia (82–85). Most of these patients have mutations in a domain of the full length NFKB2, called p100 degron (84), preventing its phosphorylation and subsequent degradation upon activation of the non-canonical NF-κB pathway (84, 86). A recent murine study showed that mutations in p100 degron underlie autoimmunity through AIRE-independent defects of thymic tolerance, which are largely mediated by changes in the thymic epithelium, including thymic medullary hypoplasia (87).

There may also be thymic stromal malfunction in patients with TTC7A defects with severe enteropathy and immune deficiency. TTC7A regulates the dynamics of the actin cytoskeleton in lymphocytes. It is also abundantly expressed on TECs (88, 89). The impact of *TTC7A* mutations on TECs has not yet been investigated, but post-mortem analysis of thymus tissue from one patient showed a disrupted thymic architecture with dysplastic changes and blurred cortico-medullary demarcation, yet preserved Hassall’s bodies. Several of these patients underwent HSCT (89, 90). The development of naïve T-cells after HSCT in one of the patients reported suggests that thymic function could be restored upon repopulation of the thymus with HSCT donor-derived thymocytes (89). This would be consistent with the notion that thymic dysfunction in these patients is mainly secondary to lymphodepletion rather than a primary TEC defect.

### Congenital Athymia in the Context of cDGS

Most commonly, impaired thymic development is associated with DGS. A triad of congenital heart disease (CHD), hypoparathyroidism and thymic hypoplasia are the hallmark features of the clinical phenotype of DGS patients (91, 92). DGS has been described in a heterogeneous group of disorders, both genetic and environmental, with variable clinical penetrance. A minority of DGS patients are diagnosed with cDGS, which is characterized by thymic aplasia and/or absent thymic function with absolute T-cell counts below 50x10^6^/L and absent mitogen responses (64). B-cell counts are within normal ranges, but T-cell mediated help for antibody production is impaired. cDGS patients thus display a combined immunodeficiency (CID) phenotype (93). Over time, a significant number of cDGS patients, develop atypical features mediated by inflammation causing erythroderma, enteropathy and lymphadenopathy. This Omenn-like picture is due to oligoclonal expansion of dysfunctional T-cells of memory phenotype (94, 95). In atypical cDGS, absolute T-cell counts may be high, but the proportion of naïve T-cells remains negligible (<5%), TCR spectratyping as well as the repertoire of the V beta TCR chains are abnormal, and mitogen responses are impaired. cDGS patients, typical and atypical, usually suffer from severe infections during infancy and, in absence of treatment by allogeneic thymus transplantation or hematopoietic stem cell transplantation (HSCT), cDGS is fatal in the first two years of life. In contrast, patients with partial DGS (pDGS), have higher absolute T-cell counts and display milder degrees of immunodeficiency, including variable susceptibility to infections and autoimmunity. Those most affected require supportive measures such as antibiotic prophylaxis (92).

cDGS is the most frequent etiology of congenital athymia. This may have a number of different causes (**Table 1**). The most common is chromosome 22q11.2 deletion syndrome (22q11.2DS) (10, 12). 22q11.2DS occurs approximately in 0.25 in 1000 births (96, 97), of which 1.5% display cDGS with athymia (98). Most deletions at chromosome 22q11.2 result in haploinsufficiency of TBX1, a transcription factor regulating key events in early thymus embryogenesis (99, 100). 22q11.2DS patients represent more than a third of the patients who underwent thymus transplantation to date (10, 12). The second most frequent disorder in both cohorts of thymus transplanted cDGS patients is CHARGE syndrome (10, 12). CHARGE syndrome is mainly due to *CHD7* variants and is found in about 1 in 8500 to 1 in 10000 births (101–103). The proportion of these infants who have cDGS is unknown, but a reported series of 280 cases did not mention immunodeficiency which is consistent with the proportion being below 1% (104). The third most frequent etiology of athymia, accounting for 10-20% of transplanted cDGS patients, is embryopathy secondary to poorly controlled maternal diabetes (10). The incidence with which this complication occurs again remains unascertained. Less frequent etiologies of DGS include other monogenic disorders and chromosomal microdeletions, as well as exposure to other fetal toxins (105, 106). Pathogenic variants in *TBX1* and *TBX2* have been reported in few patients, including some suffering from thymic hypoplasia and aplasia (12, 65, 107, 108). It is unclear whether any of the patients with partial chromosome 10p monosomy (109) suffer from complete athymia. In a report of 32 cases, 28% had a partial immunodeficiency phenotype but none had a picture compatible with athymia (110). *FOXI3* haploinsufficiency secondary to microdeletions at chromosome 2p11.2 was recently described (111), but whether this may cause complete thymic aplasia is not clear as detailed immunophenotyping of thymic output has not been published. Regardless of the underlying pathogenesis, DGS patients often have associated clinical features, such as CHD, hypoparathyroidism, typical facial dysmorphism, velopharyngeal anomalies and feeding difficulties, which prompt physicians to their diagnosis. As there are no predictive clinical factors to reliably identify the small proportion of DGS patients with athymia, it is important that upon diagnosis of DGS, patients are screened for possible severe immunodeficiency.

### Congenital Athymia in the Context of Nude SCID and OTFCS2

More infrequently than in DGS, athymia has been described in T^-^B^+^NK^+^ SCID. In classical Nude SCID, athymia is associated with *alopecia totalis* and nail dystrophy (112). This is an autosomal recessive (AR) disorder due to bi-allelic loss-of-function (LOF) mutations in *FOXN1*, a critically important transcription factor expressed in epithelial cells of the thymus, skin and nail matrix (113). Nude SCID has been reported in a small number of patients in whom FOXN1 deficiency underlies an impaired development of the thymic epithelial stroma with subsequent impaired T-cell differentiation (66, 67, 114–116). Their profound T-cell lymphopenia is marked by very low counts of naïve T-cells and recent thymic emigrants, as well as impaired T-cell proliferation responses. A handful of Nude SCID patients have been treated by HSCT, of which three, receiving transplantation from a matched sibling donor, have been reported (67, 116–118). Only one patient survived, but without regeneration of CD4^+^ naïve T-cells (117).

T^-^B^+^NK^+^ SCID has recently been described in another rare AR disorder, otofaciocervical syndrome type 2 (OTFCS2), which is caused by PAX1 deficiency and is characterized by syndromic features, including facial dysmorphism, ear and vertebral anomalies (119, 120). PAX1 is a transcription factor with a key role during thymus embryogenesis. In OTFCS2 patients from four unrelated families, bi-allelic LOF mutations in PAX1 resulted in severe T-cell lymphopenia due to altered thymus development (120). Four of these patients underwent HSCT before identification and characterization of their genetic defect, but despite full donor engraftment, T-cell immunity remained impaired. Naïve T-cells did not develop, consistent with absent thymopoiesis. Rather than treatment by HSCT, we would thus recommend referral of FOXN1^-/-^ and PAX1^-/-^ SCID patients for thymus transplantation.

### Congenital Athymia in the Context of Undefined, Novel Defects

The incidence of congenital athymia among profoundly T-cell lymphopenic patients is not known. Even though it is rare, it is becoming increasingly apparent that a non-negligible proportion of SCID patients are affected by genetically defined and undefined thymic stromal cell defects. In the Italian SCID cohort, for example, genetically defined thymic disorders were found in 6% of the patients (121). This is almost certainly an underestimation of athymic cases, as a significant number of patients with inborn errors of immunity, including SCID, do not have a genetic diagnosis despite the expanding access to next-generation sequencing (NGS) (122, 123). In a recent cohort of SCID patients, 7% of the patients did not have a genetic variant in any of the known SCID genes (124). Additionally, timely diagnosis of thymic defects is complicated by the clinical variability among affected patients and in practice, not infrequently, inborn errors of thymic development and function are missed or are only recognized after a failed attempt at T-cell reconstitution using HSCT. In such cases, thymus transplantation can be successful as a second procedure particularly if it is possible to use a thymus matching for any MHC alleles (also called human leukocyte antigen (HLA) alleles in humans) in the recipient which were mismatched with the original HSCT donor to reduce the risk that the procedure might facilitate graft-versus-host disease (GVHD) mediated by the HSCT donor cells (125). However, such “rescue” second treatments will not always be feasible and depend on the absence of severe post-HSCT morbidities. It is important therefore to discriminate between patients with hematopoietic defects, who require a HSCT, and patients with thymic defects so that, where feasible, thymus transplantation should be considered as the preferred option. The progressive introduction of newborn screening (NBS) programs for SCID in a growing number of countries should facilitate this. NBS is based on the measure of T-cell receptor excision circles (TRECs) from dried blood spots by quantitative PCR and identifies infants with low T-cell numbers, including those with non-SCID T-cell lymphopenia (126, 127). NBS is essential to improving the outcome for patients by starting prophylactic measures early on, while completing immunological and genetic investigations with the aim of proceeding with the appropriate curative treatment.

Genetically undefined T^-^B^+^NK^+^ SCID and non-SCID T-lymphopenia pose a serious therapeutic dilemma, as the underlying defects are either intrinsic to HSC differentiation, or HSC-extrinsic when secondary to impaired T-cell differentiation in the thymus. Existing *in vitro* T-cell differentiation tools that assess the intrinsic potential of HSC to differentiate into mature T-cells have been useful to address this challenge. T-cell differentiation has been pursued in *ex vivo* culture models since 2005 and makes use of co-culture systems or functionalized microbeads able to provide Notch Delta-like ligand (DLL)-1 or DLL-4 to HSC differentiating toward T-cell fate (128–130). We have taken advantage of a monolayer co-culture system of patients’ bone marrow-derived CD34^+^ cells together with OP9/DL1 stromal cells and growth factors (49). Recently, two groups reported the use of artificial thymic organoids aggregating DLL4-expressing stromal cell lines (OP9/DLL4 and MS5-hDLL4) to facilitate the differentiation of CD34^+^ cells, isolated either from the patients’ bone marrow aspirate (52) or from their peripheral blood (131). Using these three-dimensional tools, they have shown that CD34^+^ cells of two cDGS patients, one with a *TBX1* mutation and the other with a deletion at chromosome 22q11.2, had the potential to differentiate into CD3^+^ mature T-cells, in line with their disorder not affecting the HSC. Whereas, in contrast, they observed early developmental blocks when attempting the differentiation of CD34^+^ cells from SCID patients with known HSC-intrinsic defects. With the propagation of NBS programs and NGS, these tools are destined to play an essential role in assisting physicians making therapeutic decisions for undefined T-cell lymphopenia. Importantly, CD34^+^ cells from patients carrying *RAG* mutations and from one patient with a *IL2RG* mutation could also be differentiated *in vitro* into T-cell stages beyond the expected developmental block, emphasizing that further work is required to optimize the use of these assays in therapeutic management.

NBS for SCID was commenced in parts of the United States in 2008. The North American experience so far highlights that even when a thymic stromal defect is genetically and functionally confirmed early, evaluating the best therapeutic approach remains difficult in the case of rare, novel defects. This is the case, for example, in regards to the management of infants with selective T-cell lymphopenia and low TREC levels at birth who have been diagnosed with hypomorphic heterozygous *FOXN1* mutations since the introduction of NBS (132, 133). None displayed alopecia *totalis* and nail dystrophy was seen in approximately half the reported cases only. Immunological follow up showed that in most individuals, absolute CD4^+^ T-cell counts normalized over time, while CD8^+^ T-cell counts typically remained below the normal range. Most patients either displayed mild immune deficiency, mainly with recurrent respiratory infections, or remained largely asymptomatic. Yet 1 in 8 of the reported cases with *FOXN1* heterozygosity suffered a more severe degree of immune deficiency. An additional recent report highlights that a spectrum of clinical manifestations may be associated with different mutations (67). Disease severity likely depends on the residual FOXN1 protein activity. In three patients treatment was attempted with HSCT before the genetic diagnosis was made but their T-cell immunity remained impaired (132), suggesting that thymus transplantation, and not HSCT, should be considered in carefully selected FOXN1^+/-^ patients with more severe clinical phenotypes. In the mouse model, a gene dosage effect was documented for Foxn1 expression in TECs for all of the heterozygote variants. Three patients with compound heterozygosity for LOF mutations also displayed more severe clinical phenotype (67, 133). While most FOXN1^+/-^ patients can be successfully managed conservatively, a better understanding of the mechanisms of action of these mutations and of their impact on FOXN1 function is necessary to be able to predict which cases should at least be considered for potentially corrective treatment.

## Thymus Transplantation for cDGS

The process of thymus transplantation using cultured, postnatal thymus tissue has not changed much since it was first developed (9).

### Preparation of Thymus and Transplantation

Donor thymus tissue is obtained with parental informed consent from otherwise healthy infants undergoing cardiac surgery *via* median sternotomy for congenital heart disease. Removal of thymus tissue is necessary to facilitate surgery in these patients. There are increasing concerns over the potential long term consequences of very early total thymectomy (134–136) and surgeons should where possible conserve some of the thymus tissue. The preparatory process has been previously described (9).The removed thymus requires culturing for 13-20 days before transplantation in order to deplete thymocytes while preserving the thymic stroma. This process has been shown to be consistent between slices of the same thymus and between different thymus cultures (137). The lymphodepletion is incomplete with some lymphoid cell preservation showing a relative enrichment of viable CD4^+^ SP cells (12, 137). The stromal cells in contrast retain over 90% estimated viability (12, 137). Dependent on satisfactory histological evaluation and negative screening for infectious agents the cultured thymus slices are then implanted into the quadriceps muscles as previously described (138). It is important to note that the thymus donor is not tissue type matched with the recipient.

### Patients

Eligibility for thymus transplantation requires a diagnosis of cDGS based on clinical features of DGS, with or without a genetic diagnosis, associated with either extreme T-cell lymphopenia (in typical cDGS) or T-cells showing a restricted repertoire with very low naïve T-cells (< 50x10^6/L) (in atypical cDGS, often with Omenn–like features). Among transplanted cDGS patients reported in both series the proportion showing atypical features varied between one third and two thirds (10, 12). Patients with other causes of athymia can also, appropriately, be treated with thymus transplantation (discussed later in this review). They may also have an atypical (Omenn-like) phenotype (66, 114, 116).

Prior to transplantation it is important to treat or correct other co-morbidities such as congenital heart defects which might affect the clinical stability of the patient after thymus transplantation. Those with the atypical phenotype require immunosuppression while the remainder are transplanted without conditioning. This immunosuppressive treatment involves Cyclosporin A (CSA) and antithymocyte globulin (ATG) with methylprednisolone given prior to the transplantation of the allograft. The CSA is then continued post-transplantation until evidence of thymic output is achieved.

### Outcome

Overall survival has been reported as around 75% in transplanted cDGS cases (**Table 1**). Infection, often acquired prior to transplantation, was the most frequent cause of death (10–12). Following transplantation, the recipient’s bone marrow-derived hematopoietic precursors repopulate the thymic graft. It takes a minimum of four months and often longer before T-cell numbers increase with evidence of thymic output. Evidence of developing thymopoeisis can however be obtained from biopsies of transplanted tissue from as early as two months (139). Typically, the absolute numbers of T-cells achieved do not reach the normal age-related range and neither do the absolute numbers of naïve T-cells and TRECs, however there is a diverse T-cell receptor repertoire and normal T-cell proliferation in response to mitogens and normal HLA-restricted specific antigen responses (10–12). An analysis of variables associated with the procedure and the outcome in terms of T-cell and naïve T-cell numbers showed no effect of thymus dose, use of immunosuppression or chance partial HLA-matching between donor and recipient (140). ABO blood group incompatibility was associated with lower T-cell counts at 1-2 years post-transplantation but not at 3 years (11). In our unit, selected donors are blood group compatible with the athymic recipients. Even though there is no matching for HLA tissue type, the recipients’ T-cells have been shown to become tolerized to donor specific HLA antigens (141, 142). The mechanisms by which a HLA-mismatched thymic graft achieves self-tolerance remain to be determined. It is likely that host-derived hematopoietic cells, in particular DCs, can migrate into the graft and therefore contribute to HLA-TCR mediated interactions with developing thymocytes undergoing negative and positive selection. It is also possible that, akin to what has been observed after kidney and lung transplantation (143, 144), host stromal cells develop in the graft, resulting in a chimeric thymus including host fibroblasts which may contribute to direct lymphostromal interactions. Additionally if there is some serendipitous partial HLA-matching, direct TEC-thymocytes interactions may occur, regardless of host or donor origin of the cells. These different hypotheses are summarized in **Figure 1B**.

For the majority of transplanted patients in either cohort the long term outcomes of the immune recovery have not been described yet. The observations in the small numbers of reported patients with follow up of more than 3 years (up to 8 years) after thymus transplantation show sustained, even if suboptimal, T-cell counts (10, 12, 138, 145), whereas TREC levels, which correlate directly with thymic output, typically decrease and plateau over time (12). In spite of the lower than normal T-cell counts, patients develop the ability to clear both pre-existing and acquired infections. Immunoglobulin replacement therapy and antibiotic prophylaxis can be stopped. Patients can respond to vaccination with antigen-specific cell responses. Regulatory T-cells have been shown to be present in normal proportions of circulating T-cells though in subnormal absolute numbers (because of the relative T-cell lymphopenia) and these cells functioned normally in a CTLA-4 mediated endocytosis assay (12).

### Complications

Particularly in children with DGS, there may be multiple system co-morbidities which can complicate the post-transplantation course by jeopardizing the general stability required for successful engraftment of the transplant with repopulation of the new thymus by recipient-derived progenitor T-cells. These complications need to be differentiated from those potentially arising as a direct result of thymus transplantation. Furthermore, the prolonged wait before immune reconstitution is seen, means that transplanted children remain susceptible to infective and possibly to autoimmune complications as a result of their underlying condition rather than the procedure itself. Inflammatory complications may be seen after transplantation and fall into three categories. First, particularly in those not treated with ATG prior to transplantation, oligoclonal T-cell expansions and Omenn-like features may still develop. This requires the institution of CSA treatment. Secondly, with persistent infections, at the time of immune reconstitution, there may be an immune reconstitution inflammatory response (IRIS). This has been seen in those infected with BCG, rotavirus (including vaccine strain), norovirus, *Clostridium difficile*, and HHV6 ( (12) and unpublished observations). Finally, a small number of patients have been seen in whom a severe systemic IRIS occurred without an underlying identifiable driving pathogen (unpublished). These complications can be life-threatening and typically require the use of high doses of cortico-steroids, often resulting in a delay in the development of thymopoiesis.

Autoimmune complications are relatively common after thymus transplantation (**Table 1**). Early autoimmunity, prior to immune reconstitution, included hemolytic anemia (12) and renal disease has also been seen at this stage (142). Mostly these patients did well with no recurrence. Later autoimmunity predominantly involves thyroid disease but cytopenias are also common. Out of sixty transplanted patients in one series, thirteen developed thyroid disease and nine had cytopenias (11). In our series the incidence of autoimmunity may be higher than this. In most cases the autoimmune disease is either relatively easily treated, as in the case of thyroid disease, or a lasting remission can be achieved after initial therapy in the case of cytopenias. However in one small series one patient’s hemolysis required ongoing immunosuppressive treatment while one patient died of a cerebral hemorrhage complicating immune thrombocytopenia (12). The frequency of these complications suggests they occur at least in part not because of the background susceptibility as part of DGS, but because of a failure of generation of central tolerance to these particular organ systems after transplantation. The lack of HLA-matching between donor and host may be responsible due to likely impaired lymphostromal crosstalk in the thymic grafts. Coincidental partial HLA-matching within donor-recipient pairs at one or more loci was seen in all patients without autoimmune complications described in the initial report on the UK cohort (12), whereas all patients without any HLA-matching developed autoimmune complications. A trend toward less autoimmunity in a partially HLA-matched setting was not statistically significant, but the number of patients was too small (n=10) to properly address this question. Additionally achieving matching at specific loci or of specific alleles may be of importance.

## Thymus Transplantation for Non–DGS Thymic Stromal Defects

Thymus transplantation has also been shown to be the most appropriate treatment for athymia due to FOXN1 deficiency. A successful outcome after thymus transplantation in nude SCID has been reported in 2 patients. Interestingly in one of those patients reconstitution with naïve circulating T-cells was very slow, taking over twelve months (66). The long term follow up of the other patient showed sustained immune recovery, despite decreased thymic output over time (68), similar to what has been observed in transplanted cDGS patients. More recently, we have transplanted three further cases with homozygous or compound heterozygous *FOXN1* mutations (67). Two had allograft biopsies confirming the establishment of thymopoeisis of whom, one has done well and one died as a consequence of severe inflammatory complications. The third patient has not developed thymopoeisis after 17 months.

We have also very recently transplanted two athymic OTFCS2 patients, but the outcome for these cases is not yet known.

Thymus replacement therapy has not at the present time been attempted in patients with APS1. The attraction of attempting this would be to halt the inexorable progression to multisystem autoimmune complications seen in these patients. However, such an approach would have additional challenges over and above those normally encountered, including the question of competition for bone marrow precursors between the recipient’s own thymus and the transplanted tissue, the possibility of rejection and the likely presence of pre-established autoimmune problems. Furthermore, recent work has highlighted the potential benefit of early, pre-symptomatic immunomodulatory therapies that may improve the clinical outcome for these patients (73). This makes the case for a radical intervention with thymus transplantation less attractive.

## Possible Strategies for Improving the Use of Cultured Postnatal Thymic Tissue

### Improving the Pre-Transplantation Thymic Tissue Culture Process

As mentioned above, the lymphodepletion during pre-transplantation tissue culture is incomplete and viable donor T-cells are transplanted with the graft (12, 137). While it is possible that such HLA-mismatched donor T-cells could mediate GVHD, this has not been reported. On the other hand, these cells may be helpful in providing necessary lymphostromal interaction to support initial TEC homeostasis post-thymus transplantation. Whether the self-renewing lymphoid progenitors as well as other donor-derived hematopoietic cells, including dendritic cells and Tregs, survive the culture period is not known. Repercussions on TEC function have been reported with downregulation of AIRE expression in culture (146), though normal AIRE expression has been shown in biopsies taken from 2-3 months after transplantation (12). It is therefore possible that after thymus transplantation early thymopoiesis occurs in the absence of adequate AIRE-mediated negative selection, possibly contributing to failure of early deletion of autoreactive T-cells and post-transplantation autoimmunity. These observations support the need for studies addressing whether modulation of the preparatory culture protocol might improve AIRE expression and potentially reduce autoimmune complications.

### Using Cryopreserved Thymic Tissue

Thymus transplantation is a complex procedure which is only offered at 2 centers worldwide. Inevitably, access to the treatment is restricted to patients who have access to the resources to support traveling significant distances, sometimes across continents. In the future, use of pre-prepared, cryopreserved thymus tissue may make it possible for patients to be treated at institutions within their own country. We have shown that the transplantation of cryopreserved and subsequently thawed human thymic tissue into athymic mice was able to support thymopoiesis, achieving reconstitution of T-cells (147). If previously cryopreserved slices can be shown to support thymopoeisis when transplanted into athymic patients, then thymus tissue banks could be used to simplify the logistics of this treatment. It could also facilitate the use of at least partially HLA-matched tissue. In theory at least, matching at >4 loci might allow more efficient thymopoiesis in the graft, resulting in a reduced incidence of autoimmune complications. In line with this, SCID patients undergoing haploidentical HSCT procedures generally develop normal thymopoiesis in the presence of good myeloid engraftment (148).

## Future Directions for Thymus Replacement Therapy

Despite the life-saving potential of transplantation of postnatal thymus tissue described above, there are limitations to this approach. These include global accessibility, incomplete immune reconstitution and a relatively high rate of complications. New therapeutic strategies for replacing thymus function might have the potential to overcome these limitations for patients with primary athymia (**Table 2**), but possibly might also allow such treatments to be used in other clinical scenarios, such as secondary loss of thymus function through intensive chemotherapy or neonatal thymectomy, or to help tolerization after solid organ transplantation. These scenarios have been reviewed elsewhere (150).

**Table 2:** Future treatment approaches for congenital athymia (in comparison with standard treatment).

Approach	Standard thymus transplantation with cryopreserved thymus tissue	Engineered thymus using a natural scaffold seeded with expanded TEC progenitors	Engineered thymus using a natural scaffold seeded with gene-corrected iTEPCs
**ADVANTAGES**	-Improved treatment logistics -Potential for partial tissue type matching with possibly reduced autoimmunity	-Generation of large amounts of human thymus tissue -Potential for partial tissue type matching with possibly reduced autoimmunity	-Autologous procedure -Expected reduced autoimmunity
**CURRENT STATUS**	**Pre-clinical** **Mouse model** (147) Frozen & thawed human thymus tissue supporting murine T-cell development upon transplantation into nude mice	**Pre-clinical** **Mouse model** (35) Seeding of natural decellularized ECM with human thymic stromal cell progenitors by whole-organ perfusion; supporting human T-cell development upon transplantation into humanized NSG mice	**Pre-clinical** **Mouse model** (149) Small RTOCs containing (control) human iTEPCs mixed with mouse embryonic fibroblasts; supporting limited murine T-cell development upon transplantation into nude mice

In recent years, there has been a constant increase in research output aimed at producing engineered thymic stroma. The importance of the epithelium in the thymus makes it possible to apply expertise and knowledge from the epithelial stem cell field which have been used to develop successful tissue replacement strategies using cultivated stem cells. These regenerative medicine studies have already resulted in translation to the clinic for replacement of non-lymphoid organs such as the skin and the cornea (151–154). However, in contrast to the multi-layered structure of these tissues, TECs form a complex three-dimensional (3D) arrangement, which is essential for their functioning. 3D co-culture systems, referred to as re-aggregate thymus organ cultures (RTOCs), have successfully been developed using thymic stromal cells from mouse embryos (155–157). These RTOCs are able to support initial T-cell differentiation of hematopoietic progenitors *in vitro* and also *in vivo* after transplantation into athymic mice. Nevertheless, production of T-cells is still not very efficient; moreover these systems are either mouse or mouse-human hybrids and therefore the clinical applications in the context of thymic regeneration are limited due to the lack of human thymic stroma and absent instructive MHC-II expression. Attempts at culturing TECs have intensified in the last fifteen years (38, 158–160). Two groups reported significant TEC expansion in rodents and demonstrated “stemness” properties of thymic epithelial cells (158, 159).

For clinical translation, it is essential to develop new tools that can deliver TECs within an appropriate 3D structure, capable of supporting thymopoiesis. One group used artificial collagen scaffolds seeded with postnatal, gene-modified murine TECs, able to transiently express Oct4 to promote TEC expansion *in vitro* and *in vivo* (161). However, these artificial collagen scaffolds did not show efficacy in supporting thymopoiesis when transplanted into mice. Early attempts using natural ECM from decellularized mouse thymi following repopulation with freshly isolated stromal cells, lacked TEC organization and did not support *ex vivo* thymopoiesis either (162, 163). Thymus decellularization was achieved by freeze/thaw cycles and detergent-induced cell lysis (164). This method cannot be used to obtain human natural scaffolds, as it cannot be applied to the much larger human thymi. The very recent development of a novel whole-organ perfusion system has made it possible to obtain a natural decellularized ECM from human thymi, despite the lack of major vascular access (35). This has made it possible to obtain an engineered thymus after reconstitution with *in vitro* expanded human thymic stromal progenitor cells. Of note, clinically relevant numbers of expanded human TECs and other stromal progenitors were achieved within a few weeks. When transplanted into a humanized small animal model, the engineered thymi demonstrated the capacity to re-organize into a recognizable thymus with cortex and medulla, including Hassall’s bodies. This reconstituted human thymus had the ability to support thymopoiesis, both *ex vivo* and *in vivo* (35). Having overcome important obstacles, given the possibility of expanding large numbers of thymic stromal cells and of perfusing a whole thymus organ, this approach can be scaled up, thus facilitating translation into the clinic. In the future, this approach may lead to production of a large amount of human thymus stroma for transplantation. This transplanted thymus would be free of donor thymocytes, which may be beneficial, at least for any possible complications mediated by these cells in the currently used model of allogeneic thymus transplantation. The option to bank these stromal cells will also offer the possibility to achieve partial tissue type matching.

Finally, another approach for future thymus replacement therapy is to reprogram the patients’ own cells to generate TECs. Direct reprogramming of mouse fibroblasts by enforced FOXN1 expression produced TECs capable of supporting thymocyte development in the mouse (165). In another approach, by adapting protocols which successfully differentiated human embryonic stem cells into thymic epithelium (166, 167), patient-derived fibroblasts, induced to revert to a pluripotent stage of induced pluripotent stem cells (iPSCs), could be differentiated into thymic stromal progenitors (149). They could mature only upon *in vivo* transplantation into athymic nude mice and their capacity to fully instruct human HSC has not yet been demonstrated. Once optimized, this approach in combination with gene correction might make the use of autologous engineered thymus tissue possible for athymic patients, in particular in the case of monogenic defects. Potentially, thymic stromal cells induced in this way could be used to populate the decellularized human scaffolds described above. In theory, this approach could reduce the risk of autoimmune complications compared to conventional thymus transplantation.

## Concluding Remarks

Inborn errors of thymic stromal cell development and function result in immunodeficiency and autoimmunity, with the most severe thymic defects causing thymic aplasia. For athymic patients with profound T-cell lymphopenia, transplantation of cultured, post-natal thymus tissue is a life-saving treatment, achieving sufficient immune reconstitution to clear and prevent infectious complications. Failure to survive after thymus transplantation is most commonly associated with infectious complications, often in the context of pre-transplantation infections. This is expected to reduce as the use of NBS for T-cell deficiencies becomes more prevalent, facilitating earlier diagnosis and introduction of prophylaxis. Increasing access to NGS, along with the development of new tools to characterize novel thymic defects and distinguish them from primary hematopoietic defects, will also contribute to a growing number of patients and disorders to be considered for thymus transplantation. Given the suboptimal immune reconstitution and the relatively common inflammatory and autoimmune complications after thymus transplantation, there is a need to optimize treatment by developing new approaches to replacing defective thymus function. Use of cryopreserved thymus slices for transplantation may in the future make the treatment more readily available and potentially reduce the rate of complications if partial HLA-matching is confirmed as having an influence on this. In the longer term, the use of engineered thymus tissue is likely to be translated into the clinic. The ultimate aim will be to be able to use autologous cells to generate (gene-corrected) thymus stromal cells for producing engineered tissue for transplantation.

## Author Contributions

AYK, PB, and ED all conceived and co-wrote the manuscript. All authors contributed to the article and approved the submitted version.

## Funding

AK and ED are supported by *LetterOne* & Mikhail Fridman in conjunction with Great Ormond Street Hospital Children’s Charity, The Jeffrey Modell Foundation and the UK National Institute of Health Research and the Great Ormond Street Hospital Biomedical Research Centre (NIHR GOSH BRC). PB is supported by the European Research Council (ERC-Stg No. 639429), the Rosetrees Trust (M362-F1; M553), the London Advanced Therapies (C2N-AT.006), the UCL Therapeutic Acceleration Support Fund, and by the NIHR GOSH BRC.

## Conflict of Interest

The authors declare that the research was conducted in the absence of any commercial or financial relationships that could be construed as a potential conflict of interest.
